# Comparison of the volatile organic compounds in *Citrus reticulata* ‘Chachi’ peel with different drying methods using E-nose, GC-IMS and HS-SPME-GC-MS

**DOI:** 10.3389/fpls.2023.1169321

**Published:** 2023-05-17

**Authors:** Min Wang, Xue Li, Haiyan Ding, Hongping Chen, Youping Liu, Fu Wang, Lin Chen

**Affiliations:** State Key Laboratory of Southwestern Chinese Medicine Resources, Department of Pharmacy, Chengdu University of Traditional Chinese Medicine, Chengdu, China

**Keywords:** *Citrus reticulata* ‘Chachi’ peel, drying method, E-nose, GC-IMS, HS-SPME-GC-MS, volatile organic compounds

## Abstract

**Introduction:**

*Citrus reticulata* ‘Chachi’ peel (CRCP), which is named “Guangchenpi” in China, is a geographical indication product with unique flavor properties. CRCP has been used for centuries as a traditional genuine herb because of its excellent therapeutic effects. In addition, owing to its unique odor and high nutrition, it is widely used in various food preparations. Volatile organic compounds (VOCs) are regarded as an important quality marker for CRCP and are highly susceptible to effects in the drying process due to their thermal instability.

**Methods:**

In the current study, the main VOCs in CRCP were processed using different drying methods, including sun-drying, hot air drying, and vacuum-freeze drying. The VOCs were identified by the electronic nose (E-nose), gas chromatography-ion mobility spectrometry (GC-IMS), and headspace solid-phase microextraction-gas chromatography-mass spectrometry (HS-SPME-GC-MS).

**Results:**

The results showed that the CRCP dried by vacuum-freeze exhibited the highest VOCs contents and retained the richest compounds compared to those dried by other methods, which indicated that vacuum-freeze drying is the most suitable for CRCP production. Furthermore, the chemometrics analysis revealed that the primary differential metabolites of the samples generated using different drying methods were terpenes and esters.

**Discussion:**

Overall, our study would help better understand the VOCs present in CRCP with different drying methods. The outcomes of the current study would guide the drying and processing of CRCP, which is beneficial for large-scale storage and industrial production of CRCP.

## 1 Introduction

Citri Reticulatae Pericarpium (CRP) is derived from the dried mature peel of *Citrus reticulata* Blanco and its cultivars. *Citrus reticulata* ‘Chachi’ (CRC), *Citrus reticulata* ‘Dahongpao’, *Citrus reticulata* ‘Unshiu’ and *Citrus reticulata* ‘Langerina’ are the main source cultivars (Yu et al., 2018). CRC, primarily produced in Xinhui District, Guangdong Province, is a commercially essential and predominantly cultivated tangerine cultivar. It has been cultivated for more than 700 years in China. Currently, the cultivation area of CRC spans around 100,000 mu in Xinhui, and the output of its primary products exceeds 5000 tons, with nearly 100 kinds of further processed products (Liu G. W. et al., 2022). In addition, the industrial output value is 8.5 billion yuan, which generates more than 1.2 billion yuan of direct income for farmers in the region (Su et al., 2022).

CRCP is derived from the dried peel of mature fruit, considered most famous and valuable for its superior quality and therapeutic effects (Tan et al., 2015; Li et al., 2019). It has been found that the quality of CRCP is improved, with the prolonged storage period (Luo et al., 2019; Wang et al., 2022b). CRCP is not only used in Traditional Chinese medicine (TCM) but also foods, snacks, condiments, and teas, such as “Preserved Mandarin Peel”, “Ganpu Tea”, and “Tangerine Powder”, due to its unique aroma and taste (Lv et al., 2020). As a critical TCM component, CRCP has antitumor, pain and knots relief, antioxidant, and anti-neuroinflammatory activities, and has numerous health benefits for humans (Fu et al., 2017). Previous studies have illustrated several chemical components in CRCP, such as flavonoids, VOCs, alkaloids, phenolic acids, and limonoids (Zheng et al., 2021). Of these, flavonoids and VOCs are generally considered to be the main bioactive and characteristic components of CRCP(Yu et al., 2018). Though numerous studies on CRCP mainly focused on the quantification and efficacy analyses of flavonoids (Matsuo et al., 2019; Shorbagi et al., 2022), the estimation of its VOCs has received little attention. Plant odor is related to volatile components (Feng et al., 2022; Lu et al., 2022). There is a recently odor study on different varieties of peach has shown that (E, E)-2, 6-non-adienal, *γ*-decenolactone, *β*-ionone and hexyl hexanoate contributed the most to the overall aroma of peach fruit, which can distinguish different peach fruit samples well (Sun et al., 2022). VOCs are the secondary metabolites in CRCP, and there is a previous study proving that the aromatic odor of CRCP is generally imparted by volatile components (Duan et al., 2016). Previous studies have shown that the major volatile compounds in CRCP are *β*-myrcene, limonene, *β*-trans-Ocimene, *γ*-terpinene, and terpinolene. In contrast, the critical flavor compounds are hydroxymethyl furfural, hesperidin, nobiletin, and tangeretin (Cuevas et al., 2016; Li et al., 2022). Pharmacological studies demonstrated that the VOCs of CRCP exhibit anti-inflammatory and antioxidant properties. Also, these compounds confer protection against vascular damage and have a calming and suppressing effect against asthma and cough (Shen et al., 2017; Shahriari et al., 2018; Wang et al., 2018). VOCs contribute to the overall quality of CRCP and are also considered the main classifying factor between CRCP and other cultivars (Luo et al., 2019; Zheng et al., 2021). Generally, the VOCs are easily changed due to various factors, such as the harvest period or production processes. The traditional production of CRCP mainly includes five stages: fruit picking, peel opening, reverse peel, dry peel, and aging. The natural sun drying method is primarily adopted for the drying process. However, a previous study has revealed that different drying methods can have varying impacts on the aroma-related compounds of food or herbs (Yu et al., 2022). During the drying process, volatile compounds can be lost in the samples, which affects the quality and sensory properties (Xia et al., 2021). In addition, the currently used drying method may cause CRCP deterioration, such as moth-eaten or mildew, which is not conducive to storage and cannot assure the quality of CRCP. Therefore, comparing the effects of different drying methods on the VOCs in CRCP and screening out the best drying methods for CRCP production is of great significance to ensure the quality of CRCP.

VOCs estimation methods can be divided into the instrument-based and sensory-based analyses (Wu et al., 2022). Methods based on the instrument analysis include gas chromatography-mass spectrometry (GC-MS), gas chromatography-olfactometry (GC-O), gas chromatography-ion mobility spectrometry (GC-IMS), and comprehensive two-dimensional gas chromatography time of flight mass spectrometry (GC×GC-TOFMS) (Liu H. et al., 2022). GC-MS is a well-established analytical technique for studying VOCs in CRCP, which can be combined with HS-SPME. HS-SPME has been considered to be the simplest and the most direct method to obtain volatile compounds from plant (Nekoei and Mohammadhosseini, 2018). HS-SPME-GC-MS is a sensitive and comparatively simple method with easy sample preparation steps(Zhao et al., 2022). It is most frequently used for the qualitative and quantitative determination of VOCs in foods and herbs (Nekoei and Mohammadhosseini, 2017; Nekoei and Mohammadhosseini, 2018; Mohammadhosseini et al., 2021). HS-SPME-GC-MS approach was proven to be effective in estimating the effects of a season change on volatile compounds in green tea (Wang et al., 2022c), analyzing the volatile compounds of traditional Chinese sesame oil (Chen et al., 2022), and monitoring VOCs in pear during storage (Gao et al., 2022). GC-IMS is a suitable approach for separating and measuring VOCs (Wang et al., 2022d). It enables VOCs analysis of VOCs in liquid and solid samples without pretreatment (Wang et al., 2020). In addition to providing a more precise reflection of the volatile composition in a sample, this approach is faster, more accurate, and less expensive than GC-MS analysis (Rodriguez-Maecker et al., 2017; Gerhardt et al., 2018; Wang et al., 2019). GC-IMS has been used increasingly for VOCs characterization in recent years due to its ability to effectively and intuitively distinguish the differences in the samples (Ding et al., 2022). A previous study has shown that GC-IMS can identify CRCP and other varieties (Lv et al., 2020). It can also characterize the critical aromatic compounds in wine grape varieties (Cao et al., 2022).

Methods based on sensory mainly include electronic nose (E-nose) and electronic tongue. The E-nose approach, derived from various aroma-sensor technologies, has been used with increasing popularity since it allows the capture of real-time data regarding the chemical and physical nature and quality of plants (Qiu and Wang, 2015). It has been an excellent approach for identifying VOCs in the food, cosmetic, and pharmaceutical industries (Peris and Escuder-Gilabert, 2009). E-nose effectively distinguished the CRCP from different CRP cultivars (Li et al., 2019), and the flavor compounds in Nanjing water-boiled salted duck (Wang et al., 2022a). Therefore, the synergy of E-nose can be exploited with the GC-IMS and HS-SPME-GC-MS approaches for evaluating VOCs.

Previous studies have shown that the VOCs in CRCP are diverse and not homogeneous because the odor is unique (Zheng et al., 2018). Nevertheless, to our knowledge, less study or analysis has been conducted to assess the VOCs in CRCP, which is derived using different drying methods. Therefore, the current study aimed was to analyze the VOCs in CRCP with different drying methods and identify the best approach for CRCP production to effectively retain the maximum variety and the highest content of VOCs. HS-SPME-GC-MS and GC-IMS combined with chemometrics were used to compare the effects of different drying methods on the VOCs of CRCP. The outcomes of our study would help to select the best method for drying CRCP with improved quality, increased production, and enhanced product stability. Further, our work would provide a valuable reference for large-scale CRCP production.

## 2 Materials and methods

### 2.1 Samples

**Figure 1:**
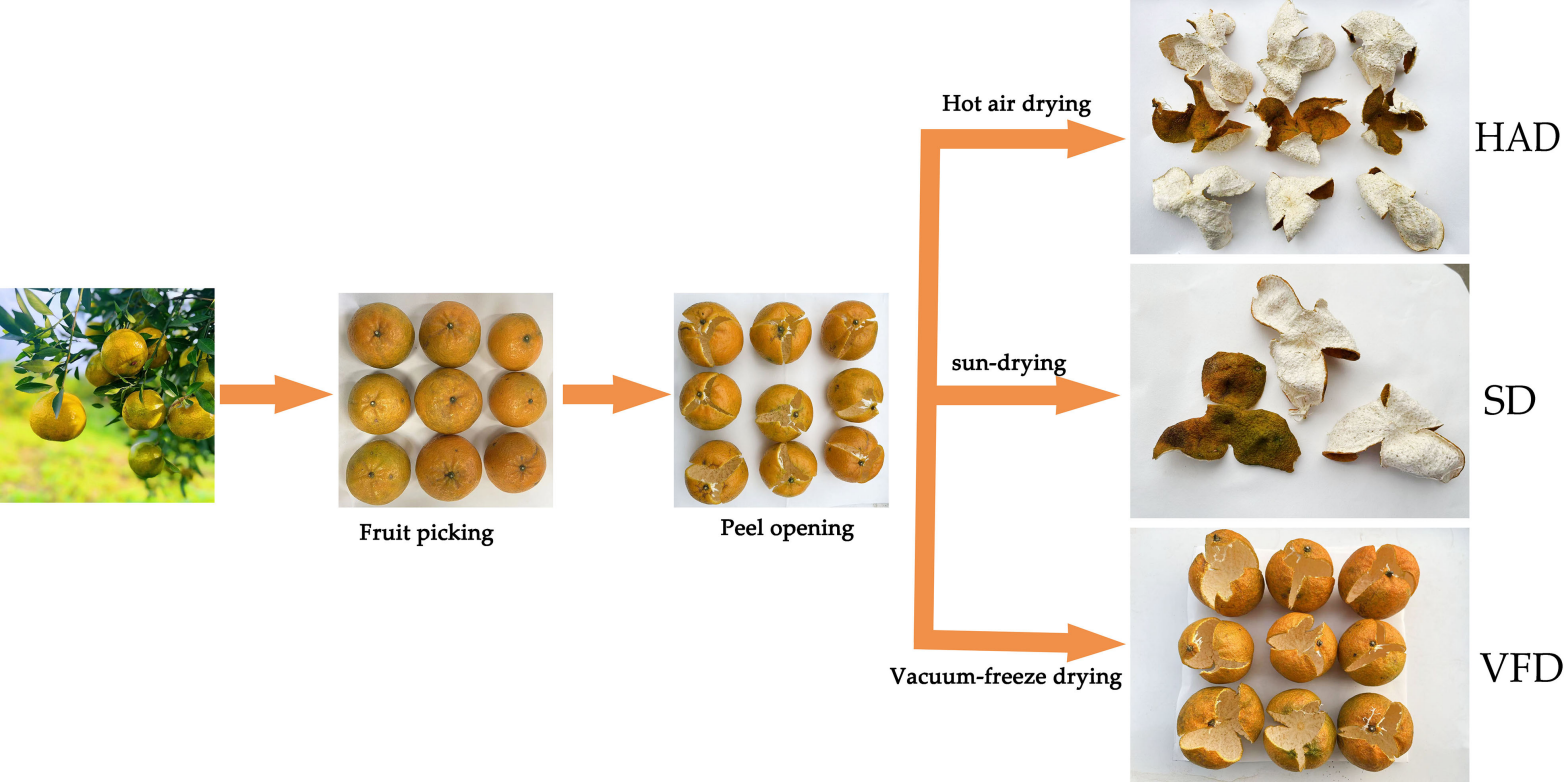
The production process of dry peel samples of *Citrus reticulata* ′Chachi′ with three different drying methods. HAD, *Citrus reticulata* ′Chachi′ peel by hot air drying; SD, *Citrus reticulata* ′Chachi′ peel by sun-drying; VFD, *Citrus reticulata* ′Chachi′ peel by vacuum-freeze drying.

CRC was collected from Sanqingwei, Sanjiang Town, Xinhui District, Jiangmen City, Guangdong Province, and authenticated by the Pharmacy College, Chengdu University of Chinese Medicine. CRCP was washed with clean water, artificially peeled, and randomly divided for different drying approaches such as sun-drying, hot air drying, and vacuum freeze-drying. The sun-dried CRCP (SD) was naturally dried in sunny weather for seven days. The hot air-dried CRCP (HAD) was dried at 60°C for eight hours in the electric blast dryer. The vacuum-freeze dried CRCP (VFD) was freeze-dried at -50°C for 24 hours in the freeze dryer. The production process of the samples is shown in **Figure 1**. After preparation, all the samples were stored in a dry place at room temperature.

### 2.2 Chemicals and reagents

The standards were purchased from BioBioPha Co., Ltd. (Yunnan, China) and Sigma-Aldrich Chemical Co., Ltd. (Missouri, USA). Normal hexane was purchased from Merck & Co., Inc. (Rahway, USA). The external reference *n*-ketones (C4-C9) for GC-MS and NaCl were purchased from Sinopharm Chemical Reagent Co., Ltd. (Beijing, China). All other chemicals used in the study were of analytical grade.

### 2.3 E-nose analysis

E-nose analysis was performed using an 8 MEMS-MOS E-nose (Isensortalk Co., Ltd. Beijing, China). The working principle of E-nose is shown in **Figure S1**. In the testing process, the samples are detected by the odor analyzer, as the sensors have different responses to the odor of different samples. The samples are defined by the response values of the sensors, and then the samples are analyzed by the corresponding analysis software to distinguish and identify the samples. First, the samples were ground into powder with a mortar at room temperature. For E-nose analysis, 2.0 g of the sample was placed into a 20-mL headspace vial. Two probes were inserted into the headspace vail, one connected to the sensor chamber and the other to a charcoal filter. Each sample was run in triplicates, and data were obtained from the sensor array after 60s injection to determine the stability of the sensor signals. Data from 55s to 58s were taken for subsequent statistical analysis. After sample analysis, the system was purified with filtered clean air for 120 s to re-establish the instrument baseline. All samples were tested at room temperature.

### 2.4 GC-IMS analysis

In this study, GC-IMS (FlavourSpec^®^, G.A.S., Germany) was used to analyze VOCs in CRCP. **Figure S2** shows the working principle of GC-IMS. During the detection, the sample enters the instrument with carrier gas. According to the red dotted line, it first goes through the primary separation of the gas chromatographic column, and then enters the ion migration tube. After ionization in the ionization zone, the molecules under test migrate to the Faraday disk under the action of electric field and reverse drift gas for detection, realizing the secondary separation. Firstly, all the samples were cut into small pieces with scissors at room temperature. Then, 1.0 g of the sample was accurately weighed into a 20-mL headspace vial and incubated at 60°C and 500 rpm for 20 min in the shaker. The volume of the automatic headspace injection was 300µL and the temperature of the injection needle was 85°C. Then the volatile compounds were subjected to a chromatographic column (FS-SE-54-CB-1, 15 m, ID: 0.53 mm) for separation at 60°C for 30 min. Each sample was measured three times in parallel. The temperature of the column and IMS was 60°C and 45°C, respectively. High-purity nitrogen (purity 99.99%) was used as the carrier and drift gas. The flow rate of the drift gas was set at 150 mL/min. The programmed flow rate of the whole phase was as follows: 2 mL/min for 2 min, 10 mL/min for 8 min, 100 mL/min for 10 min, and 150 mL/min for 10 min. The total analysis time was 30 min. The VOCs were identified based on the RIs of standard substances in the GC-IMS library (G.A.S.).

### 2.5 HS-SPME-GC-MS analysis

In this study, all the samples were ground at room temperature and vortexed to mix well. About 1.0 g of each sample was weighed in 20-mL headspace vials. NaCl-saturated solution and 10 µl of internal standard solution were added to each vial. The samples were mixed by shaking for 5 min at a constant temperature of 100°C. Subsequently, 50/30 µm DVB/CAR/PDMS SPME fibers (Agilent) were inserted into the headspace above the samples in the vails for extraction for 15 min at 100°C. After extraction, the samples were resolved at 250°C for 5 min and GC-MS analysis was performed. Each sample was measured three times in parallel.

The GC-MS system comprised an Agilent 7890B gas chromatograph and an Agilent 7000D series mass spectrometer (Agilent). High-purity Helium (purity 99.999%) gas was passed at 1.2 mL/min through the Agilent DB-5MS capillary column (30 m × 0.25 mm × 0.25 µm). The volatile compounds were first desorbed from the SPME fiber by heating at 250°C for 3.5 min. The heating procedure of the column was as follows: a temperature of 40°C was maintained for 3.5 min; an increase to 100°C at a rate of 10°C/min; an increase to 180°C at a rate of 7°C/min; further an increase to 280°C at a rate of 25°C/min; and finally held for 7 min. Mass spectrometer (MS) recorded in electron impact (EI) ionization mode at 70 eV. The quadrupole mass detector, ion source, and transfer line temperatures were set at 150, 230 and 280°C, respectively. The ion monitoring (SIM) mode was used to identify and quantify analytes.

### 2.6 Statistical analysis

All experiments were performed in triplicates. For GC-IMS, the VOCal was used for viewing analytical spectra and quantifying the data. The built-in NIST and IMS databases were used for qualitative analysis of VOCs. The Reporter plug-in Laboratory Analytical Viewer (LAV) was used to compare the differences in GC-IMS chromatograms of the samples. For visually and quantitatively comparing the differences in VOCs of the samples, the Gallery Plot plug-in was used for fingerprint comparison. For HS-SPME-GC-MS, the Qualitative Analysis Workflows B.08.00 was used to open and browse the raw data for qualitative analysis. Qualitative and quantitative mass spectrometric analysis of the metabolites of the samples was based on the MWGCSIM1.0 database. MassHunter was used to integrate and corrected chromatographic peaks. The coefficient of variation was used to reflect the degree of data dispersion. Principal component analysis (PCA), hierarchical cluster analysis (HCA), orthogonal partial least squares discriminant analysis (OPLS-DA), Pearson′s correlation coefficient, and heatmap analyses were processed by R Project.

## 3 Results

### 3.1 Comparative analysis of CRCP by E-nose

**Figure 2:**
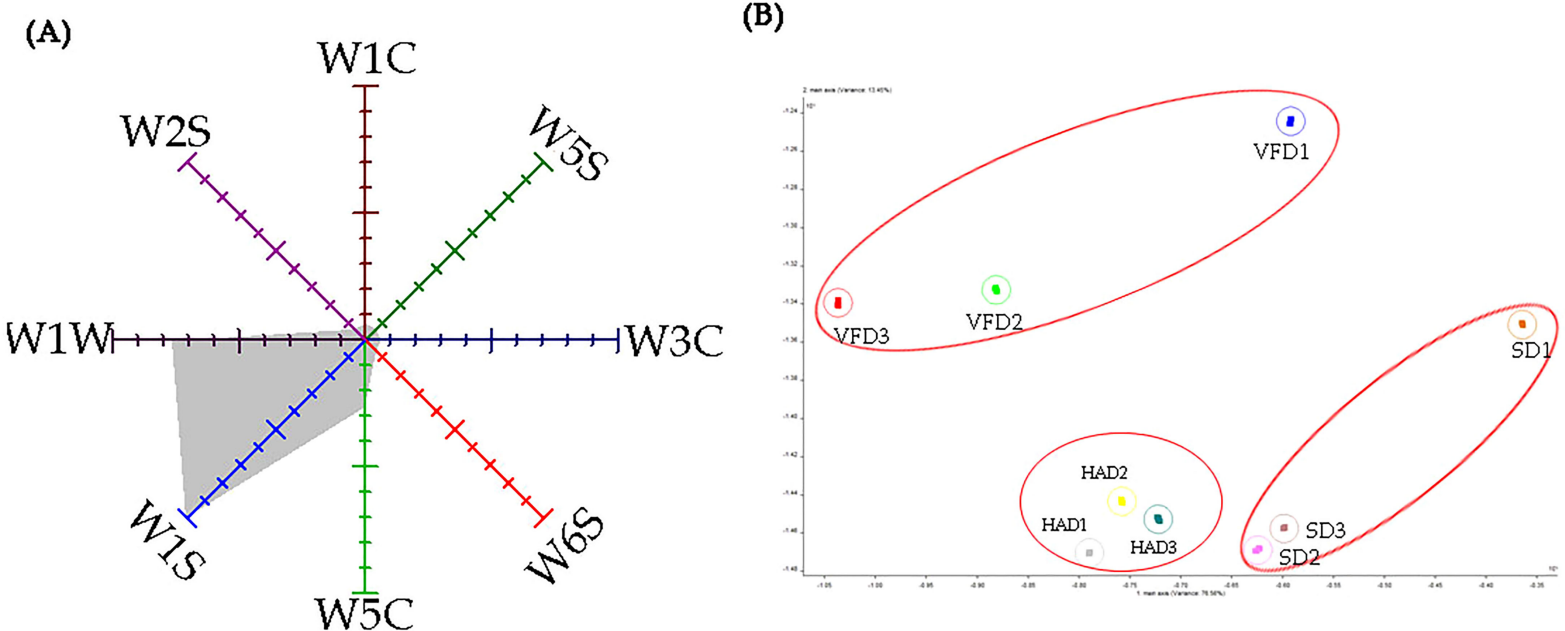
**(A)** The radar chart of the E-nose response data; **(B)** The LDA of the E-nose response data.

**Figure 2A** depicts that W1S, W1W, and W5C sensors exhibited more robust responses to VOCs in the samples. This observation indicates that the CRCP might have a higher abundance of methane, broad-methane, broad-range compounds, sulfur compounds, terpenes, organic sulfur compounds, arom-aliph, alkanes, aromatic compounds, and less polar compounds. However, E-nose is not completely sensitive to aromatic substances in CRCP. In addition, it cannot identify these substances qualitatively or quantitatively. Therefore, the E-nose can only represent a part of the aroma characteristics of CRCP.

The linear discriminant analysis (LDA) maximizes the interclass variance and minimizes the intraclass variance, i.e., reduces the differences within the classes while expanding the variations between different classes (Gerhardt et al., 2019; Xu et al., 2020; Carrillo-Gómez et al., 2021). Thus, the LDA method was used to distinguish the CRCP produced using different drying methods. As shown in **Figure 2B**, the variance contribution rates of LD1 and LD2 were 76.56% and 13.45%. The distance between the samples was relatively far, indicating that the samples were significantly different from each other. The findings revealed that CRCP with different drying methods has significant differences, which can be identified and compared further.

### 3.2 Qualitative analysis of VOCs by GC-IMS

**Figure 3:**
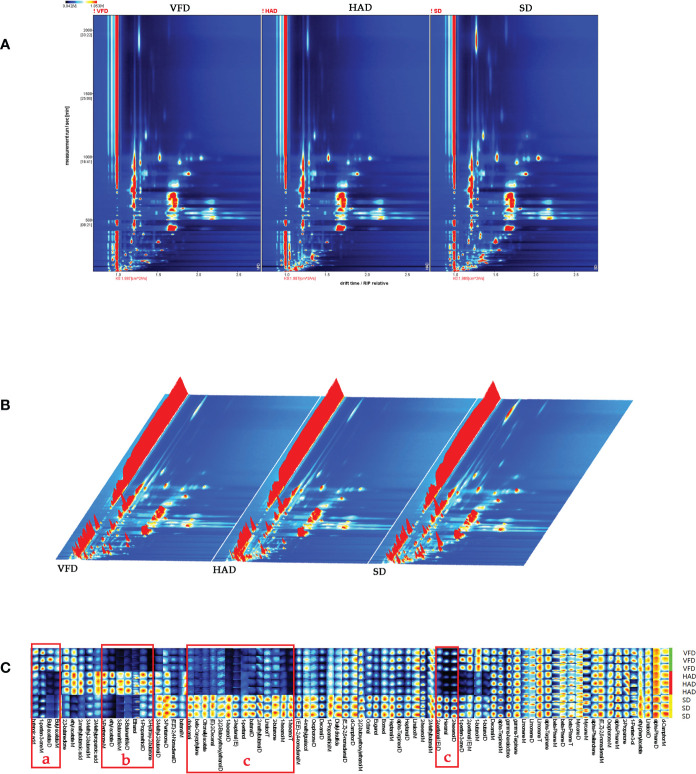
**(A)** Two-dimensional chromatogram results of VOCs in CRCP with different drying methods; **(B)** Three-dimensional chromatogram results of VOCs in CRCP with different drying methods; **(C)** The VOCs fingerprint of CRCP with different drying methods.

Unlike GC-MS analysis, which relies on the chemical morphology for analyte identification, the qualitative approach of GC-IMS is based on the two-dimensional (2D) separation of component analytes. A GC-IMS topographic map, composed of many 2D maps, acts as a global fingerprint of VOCs in the samples(Gerhardt et al., 2018). Numerous spots on a 2D topographic plot represented the VOCs of CRCP samples. As shown in **Figure 3A**, the abscissa and the ordinate are the drift and retention times, respectively. The red vertical line on the left side of the spectrum signifies the reactive ion peak (RIP), which could be used as the standard to determine the activity of the substance. Blue is the image′s background color, and the Ko is 1.987 cm^2^/Vs. The spots with different color shades represented different concentrations of each substance. In contrast, the color intensity signified the VOC concentrations, with white dots indicating a lower concentration and red dots indicating a higher concentration(Tian et al., 2020). The color strength represented the concentration. The drift time range of 1.0-2.0ms and the retention time with the 2000s were kept. The VOCs of CRCP were determined by combining retention indexes, retention time, drift times, and Ko based on the NIST database. Based on the 2D topographic plot of VFD, many red spots with high concentration were observed in the plots of HAD and SD. Especially, the maximum number of red spots in the 2D topographic plot of SD indicated that the concentration of VOCs in HAD and SD was higher than that in VFD.

A three-dimensional (3D) topographic plot was established to observe the difference directly. As shown in **Figure 3B**, the x-axis, y-axis, and z-axis of the 3D topographic plot represented the separated ions′ drift time, the detected substances′ retention time, and the signal strength of ionic compounds, respectively. We observed that the ion peak distribution of the three groups of samples is very similar, but the height of the peak signal is different. A comparison with the 3D topographic plot of VFD further revealed that the 3D topographic plot of HAD and SD have more and higher peaks.

Fingerprints can be used to distinguish closely related substances effectively. While comprehensively and visually analyzing the differences in the VOCs composition of three CRCP samples, the software selected the VOCs signal peaks in the spectra of each sample to form the fingerprints. **Figure 3C** represents the fingerprint of VOCs of CRCP samples produced with different drying methods. The results revealed that the CRCP samples with different drying methods had their characteristic peak regions and common regions. In **Figure 3C**, part “a” represents the characteristic VOCs of VFD, which mainly includes butanoic acid and butyl acetate. Part “b” indicates the characteristic VOCs of HAD, which majorly includes 1-propanethiol, 2-methylbutanoic acid, 2-methylpropanoic acid, 3-butenenitrile, 3-hydroxy-2-butanone, 3-methyl-2-butenal, 3-pentanone, ethanol, ethyl acetate. Part “c” represents the characteristic VOCs of SD, which includes (E)-2-decenal, (E, Z)-2,6-nonadienal, (E, E)-2,4-nonadienal, 2-heptenal(E), octanal, 2-methylbutanal, butanal, dodecanal decanal, 1-hexanol, 1-pentanol, 2-(2-butoxyethoxy) ethanol, 4-methylguaiacol, borneol, eugenol, linalool, oxophorone, 2-butanone, beta-caryophyllene, citronellyl acetate, D-camphor, diallyl disulfide, and a large number of other substances.

A comprehensive investigation of the topographic plots and fingerprints of various VOCs highlighted the necessity and importance of an in-depth analysis of the data from GC-IMS. GC-IMS detected about 101 volatile compounds. However, as shown in **Table 1**, only 82 compounds were identified, including 20 alcohols, 21 aldehydes, three sulfur compounds, three acids, 14 terpenes, nine ketones, seven esters, and five other compounds. The VOCs of CRCP with the three drying methods were analyzed and compared, and it was found that the VOCs of these three groups were significantly different. The main common compound was terpene, except for the characteristic compounds present at higher concentrations in each group. It was also observed that the lowest VOCs were in VFD, while the maximum number and highest concentration of VOCs were found in SD.

**Table 1:** VOCs in CRCP with different drying methods by GC-IMS.

Class	Compound	CAS#	Formula	RI ^1^	Rt ^2^	Dt ^3^
Alcohol	Ethanol	64-1-75	C2H6O	523.5	104.724	1.04805
	1-butanol M ^4^	71-36-3	C4H10O	677.4	171.576	1.17932
	1-butanol D ^5^	71-36-3	C4H10O	676.9	171.311	1.37961
	1-Penten-3-ol	616-25-1	C5H10O	702.1	186.453	0.94575
	1-pentanol	71-41-0	C5H12O	780.5	244.293	1.25153
	2-hexenol M	2305-21-7	C6H12O	867.3	332.23	1.18322
	2-hexenol D	2305-21-7	C6H12O	868.5	333.695	1.51696
	1-hexanol M	111-27-3	C6H14O	892	363.121	1.32625
	1-hexanol D	111-27-3	C6H14O	889.9	360.381	1.5759
	1-hexanol T ^6^	111-27-3	C6H14O	888.8	359.011	1.63792
	2-(2-butoxyethoxy) ethanol M	112-34-5	C8H18O3	1188.2	1083.576	1.29894
	2-(2-Butoxyethoxy) ethanol D	112-34-5	C8H18O3	1188.7	1085.725	1.8335
	Linalool M	78-70-6	C10H18O	1100.8	782.338	1.69804
	Linalool D	78-70-6	C10H18O	1099.7	779.281	1.74952
	Linalool T	78-70-6	C10H18O	1099	777.244	2.24498
	alpha-Terpineol M	98-55-5	C10H18O	1153.4	951.863	1.29472
	alpha-Terpineol D	98-55-5	C10H18O	1153.4	951.863	1.79252
	Borneol	507-70-0	C10H18O	1162.7	985.271	1.87812
	4-methylguaiacol	93-51-6	C8H10O2	1235.2	1290.935	1.18627
	Eugenol	97-53-0	C10H12O2	1342	1921.288	1.27755
Aldehyde	butanal M	123-72-8	C4H8O	583.1	126.081	1.10862
	butanal D	123-72-8	C4H8O	582.6	125.853	1.28151
	2-Methylbutanal M	96-17-3	C5H10O	660.3	162.034	1.16596
	2-methylbutanal D	96-17-3	C5H10O	664.7	164.42	1.40231
	2-pentenal (E) M	1576-87-0	C5H8O	760.2	227.652	1.10523
	2-pentenal (E) D	1576-87-0	C5H8O	760.2	227.652	1.3608
	3-Methyl-2-butenal M	107-86-8	C5H8O	791.2	253.605	1.08773
	3-Methyl-2-butenal D	107-86-8	C5H8O	792.9	255.186	1.35507
	Hexanal	66-25-1	C6H12O	809.7	270.687	1.56489
	Heptanal M	111-71-7	C7H14O	915.4	395.238	1.32879
	Heptanal D	111-71-7	C7H14O	913.9	393.04	1.69981
	2-heptenal (E)	18829-55-5	C7H12O	973.8	489.048	1.2595
	Octanal	124-13-0	C8H16O	1028.5	598.302	1.82942
	(E, Z)-2,6-nonadienal M	557-48-2	C9H14O	1128.9	868.822	1.37581
	(E, Z)-2,6-nonadienal D	557-48-2	C9H14O	1127.1	863.094	1.89839
	Decanal M	112-31-2	C10H20O	1164.6	992.252	1.5374
	Decanal D	112-31-2	C10H20O	1164.3	991.178	2.05885
	(E, E)-2,4-nonadienal M	5910-87-2	C9H14O	1207.2	1162.813	1.36453
	(E, E)-2,4-Nonadienal D	5910-87-2	C9H14O	1207.9	1166.022	1.90905
	(E)-2-Decenal	3913-81-3	C10H18O	1285	1553.832	1.45774
	dodecanal	112-54-9	C12H24O	1353.8	2007.827	1.65216
Ester	ethyl acetate M	141-78-6	C4H8O2	622.2	142.952	1.0992
	ethyl acetate D	141-78-6	C4H8O2	621.6	142.687	1.33821
	Butyl acetate M	123-86-4	C6H12O2	816.2	276.945	1.24104
	Butyl acetate D	123-86-4	C6H12O2	815.6	276.4	1.61883
	gamma-hexalactone	695-06-7	C6H10O2	1086.2	740.983	1.1942
	ethyl phenylacetate	101-97-3	C10H12O2	1235	1289.86	1.29632
	Citronellyl acetate	150-84-5	C12H22O2	1302.6	1659.3	1.45864
Ketone	2-Propanone	67-64-1	C3H6O	541.3	110.601	1.12169
	2,3-butanedione	431-03-8	C4H6O2	600.8	133.411	1.16329
	2-butanone	78-93-3	C4H8O	608.6	136.791	1.24761
	1-penten-3-one M	1629-58-9	C5H8O	687.4	177.406	1.0765
	1-penten-3-one D	1629-58-9	C5H8O	688.7	178.201	1.31284
	3-Pentanone M	96-22-0	C5H10O	700.1	185.224	1.10919
	3-Pentanone D	96-22-0	C5H10O	698.4	184.143	1.35795
	Oxophorone M	1125-21-9	C9H12O2	1115.9	827.854	1.33086
	Oxophorone D	1125-21-9	C9H12O2	1116.5	829.725	1.84344
Terpenes	alpha-Pinene M	80-56-8	C10H16	948.6	445.976	1.67808
	alpha-Pinene D	80-56-8	C10H16	949.2	447.025	1.73103
	beta-Pinene M	127-91-3	C10H16	993.1	524.956	1.64321
	beta-Pinene D	127-91-3	C10H16	991.2	521.248	1.72546
	beta-Pinene T	127-91-3	C10H16	990.8	520.506	2.19066
	Myrcene M	123-35-3	C10H16	1011.6	562.037	1.72546
	Myrcene D	123-35-3	C10H16	1011.6	562.037	2.16496
	alpha-Phellandrene	99-83-2	C10H16	1021	581.757	1.68895
	alpha-Terpinene	99-86-5	C10H16	1034.6	611.873	1.72619
	Limonene M	138-86-3	C10H16	1050.7	649.547	1.65606
	Limonene D	138-86-3	C10H16	1048.3	643.614	1.72546
	Limonene T	138-86-3	C10H16	1049.2	645.839	2.18809
	gamma-Terpinene	99-85-4	C10H16	1066.6	689.045	1.70481
	beta-Caryophyllene	87-44-5	C15H24	1322.6	1787.542	1.43563
Acid	2-Methylpropanoic acid	79-31-2	C4H8O2	764.4	230.926	1.15807
	butanoic acid	107-92-6	C4H8O2	786.8	249.723	1.16201
	2-methylbutanoic acid	116-53-0	C5H10O2	843.1	304.723	1.21017
Sulfur compounds	1-Propanethiol M	107-03-9	C3H8S	643.7	153.378	1.17294
	1-Propanethiol D	107-03-9	C3H8S	643.7	153.378	1.36195
	Diallyl disulfide	2179-57-9	C6H10S2	1114.2	822.419	1.19572
others	3-Butenenitrile M	109-75-1	C4H5N	643.9	153.474	1.12412
	3-Butenenitrile D	109-75-1	C4H5N	643.3	153.202	1.24375
	3-Hydroxy-2-butanone	513-86-0	C4H8O2	730.7	205.546	1.33384
	d-Camphor M	464-49-3	C10H16O	1127.4	864.049	1.33526
	d-Camphor D	464-49-3	C10H16O	1128	865.958	1.85559

^1^Represents the retention index calculated using n-ketones C4-9 as external standard.

^2^Represents the retention time in the capillary GC column.

^3^Represents the drift time in the drift tube.

^4^Represents the monomer of the substance.

^5^Represents the dimer of the substance.

^6^Represents the trimer of the substance.

### 3.3 Identification analysis of VOCs using the HS-SPME-GC-MS

**Figure 4:**
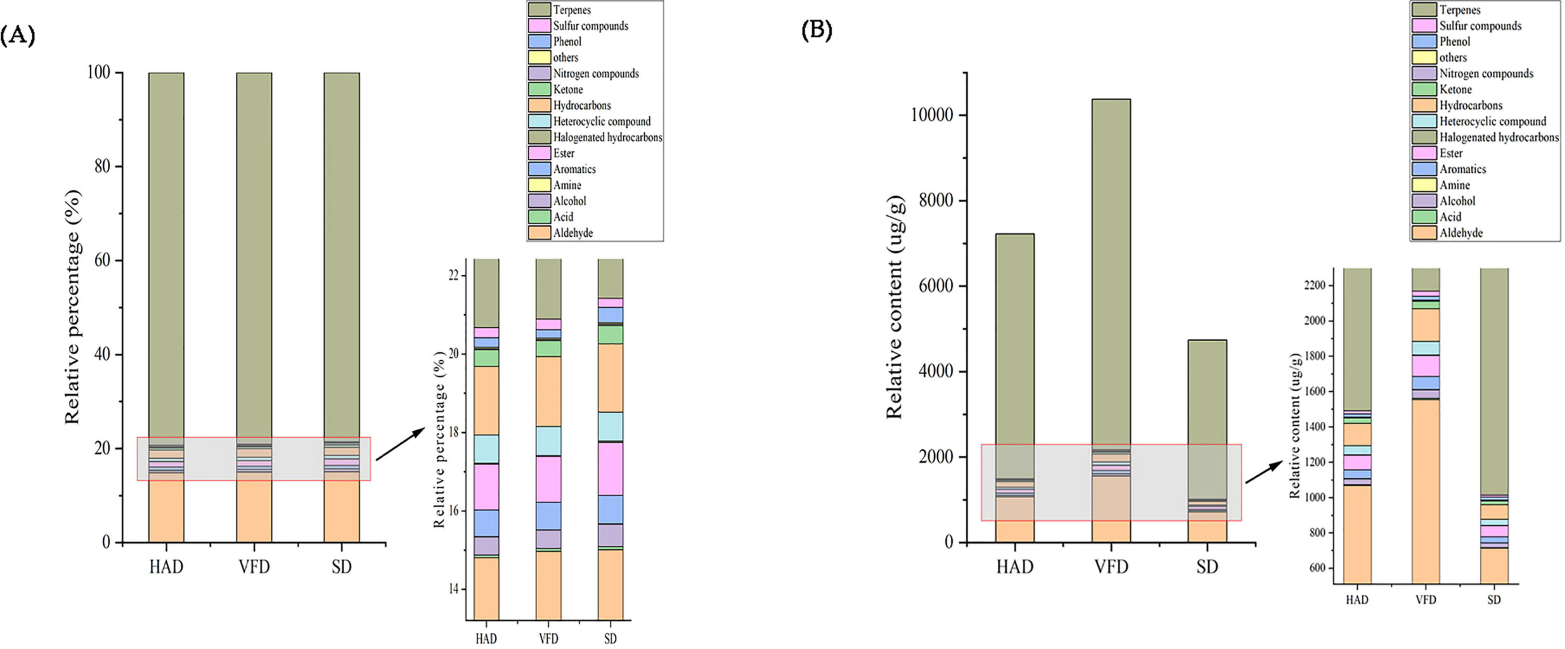
**(A)** Histogram of relative percentage of different categories of VOCs in CRCP; **(B)** Histogram of relative content of different categories of VOCs in CRCP.

**Figure 5:**
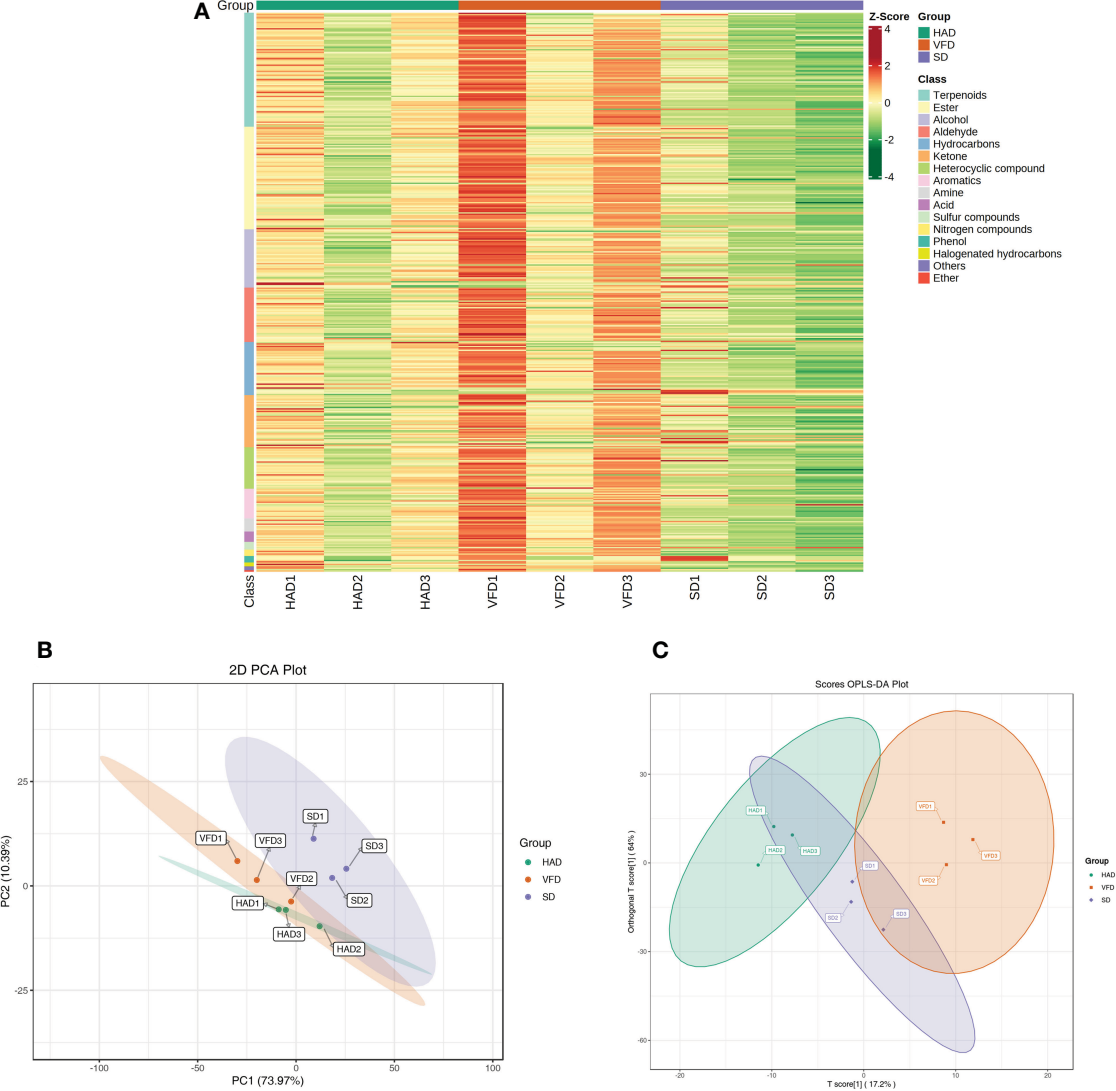
**(A)** Heat map of VOCs content for CRCP with different drying methods; **(B)** PCA scores plot of VOCs content for CRCP with different drying methods. **(C)** OPLS-DA scores plot of VOCs content for CRCP with different drying methods.

A total of 431 metabolites were detected using the GC-MS detection platform and the MWGCSIM1.0 database. **Table S1** represents 41 hydrocarbons, 24 aromatics, 79 esters, 88 terpenes, 45 alcohols, 40 ketones, 32 heterocyclic compounds, 42 aldehydes, five nitrogen compounds, ten amines, eight acids, six sulfur compounds, five phenols, three halogenated hydrocarbons, and three other compounds identified in all CRCP samples. Total ion chromatogram (TIC) overlapping map of quality control sample and TIC map of mixed sample showed in **Figure S3**. All substances were quantified with the internal standard as a reference, and the contents of various VOCs in CRCP with different drying methods are shown in **Figure 4**. The quantitative results depicted that the content of all VOCs in VFD was higher than that in the other two groups. The specific compounds in the three sample groups were analyzed, and the results of the highest ten VOCs were shown in **Table 2**, and their chromatographic integration diagrams are shown in **Figure S4**. We generated a heatmap for more intuitive observation, as shown in **Figure 5A**. The cluster heat map represented the VOC concentration in the samples. The content of VOCs in CRCP with different drying methods was significantly different, and the total concentration of VOCs in VFD was markedly higher than that in SD and HAD.

**Table 2:** The top ten VOCs with higher content in CRCP.

Compounds	Class	CAS	Formula	Aroma/Odor description
trans-.beta.-Ocimene	Terpenes	3779-61-1	C10H16	Sweet, herbal
Alpha-Ocimene	Terpenes	502-99-8	C10H16	Fruity, floral
1,5-Cyclooctadiene, 3,4-dimethyl-	Terpenes	21284-05-9	C10H16	
(E)-sabinene hydrate	Terpenes	17699-16-0	C10H18O	Woody, balsam
(Z)-sabinene hydrate	Terpenes	15537-55-0	C10H18O	Balsam
BenzAldehyde, 4-methyl-	Aldehyde	104-87-0	C8H8O	Sweet, spicy, cinnamon, fruity, bitter almond, cherry, deep phenolic
BenzAldehyde, 3-methyl-	Aldehyde	620-23-5	C8H8O	Sweet, fruity, bitter almond, cherry and tropical nutty
BenzAldehyde, 2-methyl-	Aldehyde	529-20-4	C8H8O	Cherry
.alpha.-Pinene	Terpenes	80-56-8	C10H16	Intense woody, piney and terpy with camphoraceous and turpentine note, herbal, spicy and slightly tropical nuances
Bicyclo[3.1.1]hept-2-ene, 3,6,6-trimethyl-	Terpenes	4889-83-2	C10H16	

PCA analysis of the whole sample can preliminarily estimate the overall differences in metabolites between the groups and the amplitude of variation between the samples within the group. Based on the PCA results, it can be understood whether there is a tendency for metabolome separation between groups and whether there are differences between sample groups (Chen et al., 2009). To further compare and distinguish three drying methods, PCA analysis was performed. **Figure 5B** demonstrates the PCA scores plot of the mean VOCs contents of CRCP with three drying methods measured by HS-SPME-GC-MS. PCA scores plot revealed that about 84.36% of the total variance is explained by the first two principal compounds, among which PC1 and PC2 accounted for 73.97% and 10.39%, respectively. PCA score plots showed significant differences in the VOCs among the three groups, and the short distance between three parallel samples indicated the repeatability of the data. Altogether, PCA revealed clear separation and good repeatability among the three groups.

**Figure 6:**
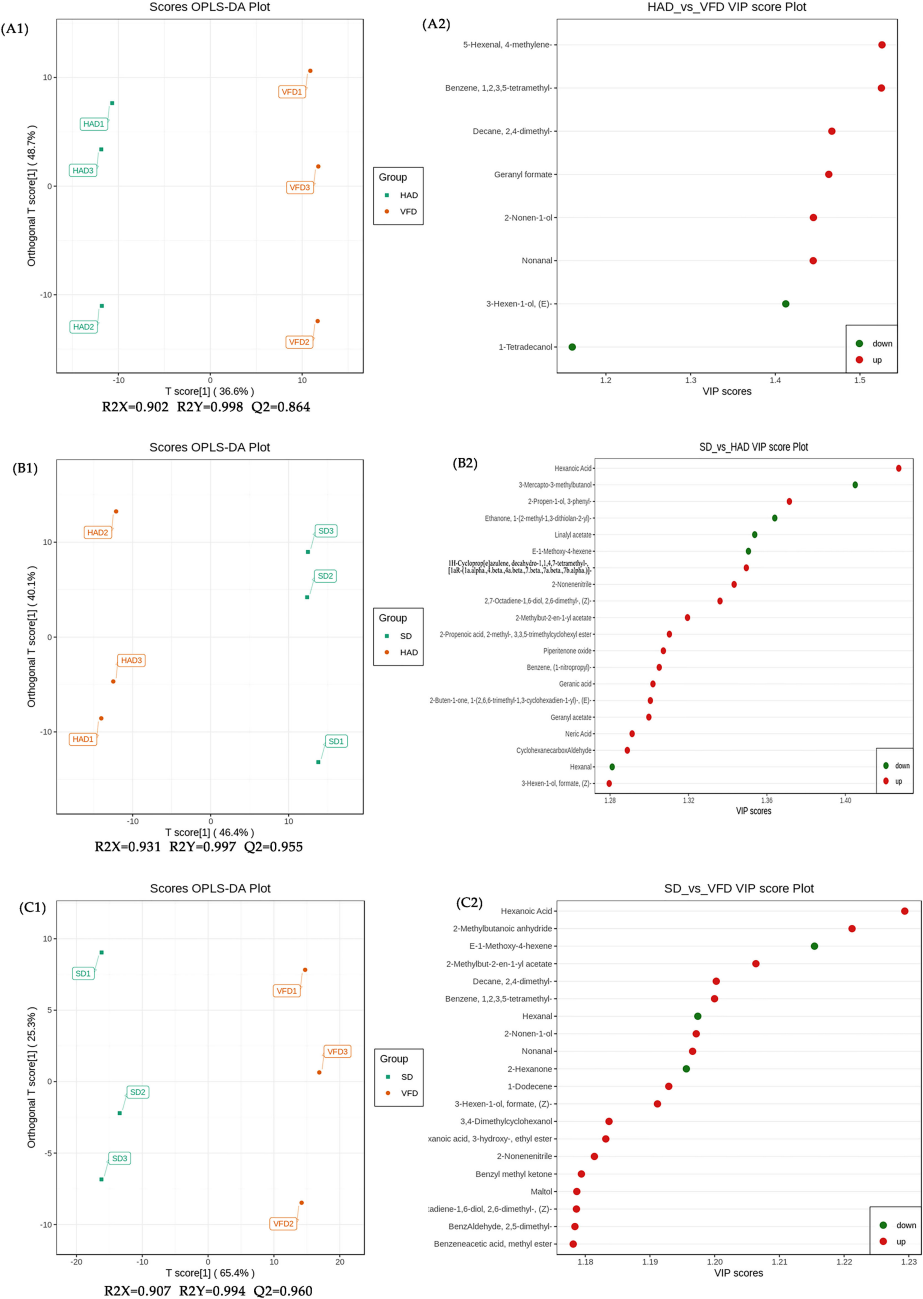
Scores plots (A1, B1, C1) and VIP plots (A2, B2, C2) of the OPLS-DA of VOCs in CRCP with different drying methods by HS-SPME-GC-MS. (A1, A2): HAD vs. VFD; (B1, B2): SD vs. HAD; (C1, C2): SD vs. VFD.

The metabolic group data were analyzed using the OPLS-DA model, and the scores of each subgroup were plotted further to demonstrate the differences between each subgroup(Thevenot et al., 2015). **Figure 5C** shows CRCP with different drying methods could be distinguished. The score plots and flavor variables [variable importance projection (VIP) > 1.0] of each group were drawn using the OPLS-DA model to further explain the flavor determiners between CRCP with different drying methods (**Figure 6**). While comparing HAD to VFD samples, 4-methylene-5-Hexenal, 1,2,3,5-tetramethyl-Benzene, and 2,4-dimethyl-Decane showed a more significant effect in discrimination. The main differentials between SD and HAD samples were dimethyl Hexanoic Acid, 3-Mercapto-3-methylbutanol, and 3-phenyl-2-Propen-1-ol. The difference between SD and VFD samples was because of the most apparent compounds: Hexanoic Acid, 2-Methylbutanoic anhydride, and E-1-Methoxy-4-hexene.

**Figure 7:**
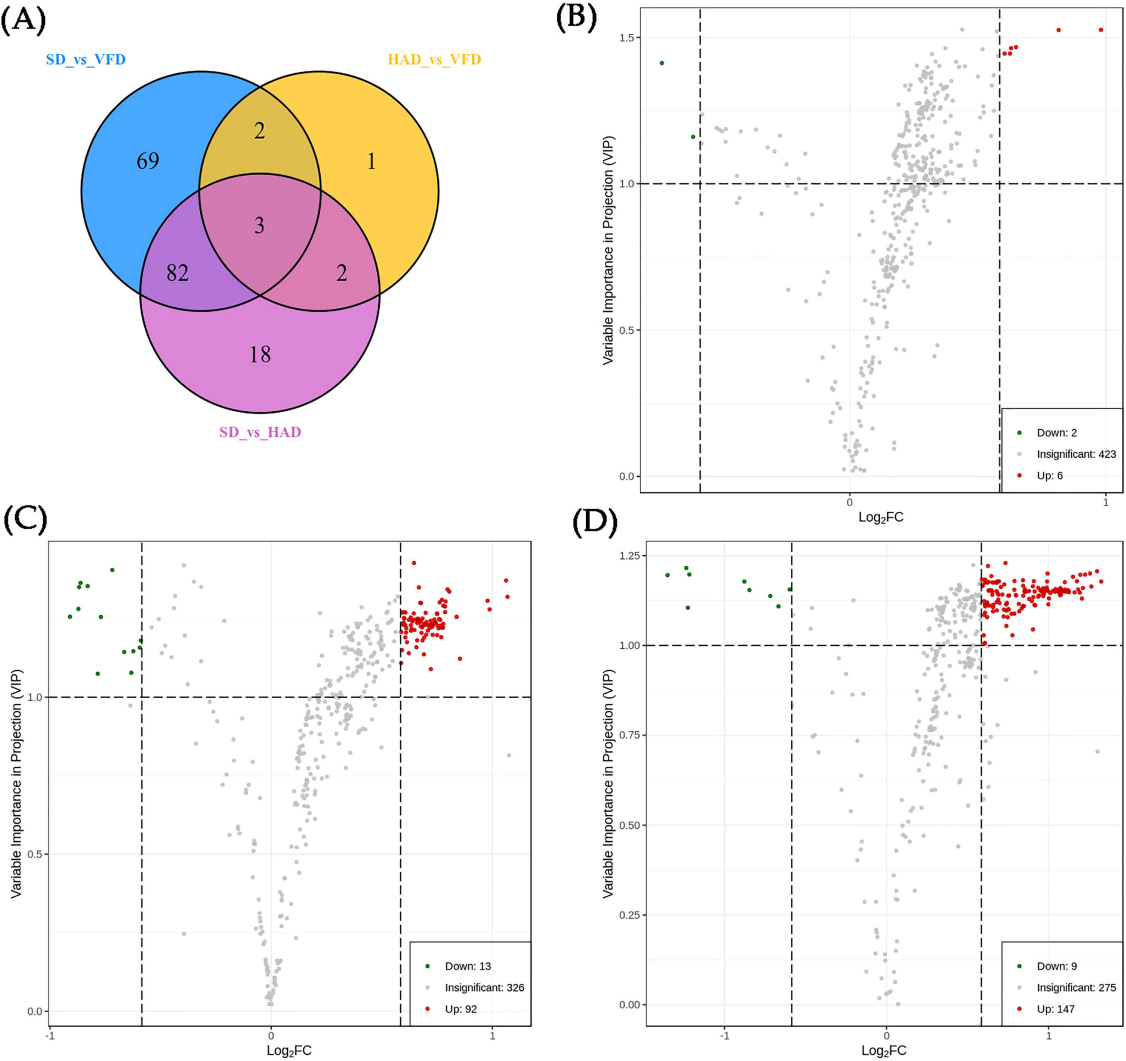
**(A)** The Venn Diagram of the differential metabolites of each group; **(B)** Volcanic plots of differential metabolites for HAD vs. VFD; **(C)** Volcanic plots of differential metabolites for SD vs. HAD; **(D)** Volcanic plots of differential metabolites for SD vs. VFD.

The PCA and OPLS-DA analyses showed that HS-SPME-GC-MS could efficiently distinguish CRCP with different drying methods. Therefore, differential metabolites can be screened to compare three drying methods for CRCP production further. Differential metabolites were screened by the VIP of the OPLS-DA model combined with fold change (FC). The screening criteria were VIP≥1, FC≥1.5, and FC ≤ 0.67. The final results of differential metabolite screening are shown in **Table S2**, which revealed eight different metabolites in HAD compared to VFD, two of which were down-regulated while six were up-regulated. There were 105 different metabolites in SD and HAD, among which 13 were down-regulated while 92 were up-regulated. There were 156 different metabolites in SD and VFD, of which nine were down-regulated while 147 were up-regulated. The Venn diagram of differential metabolites is shown in **Figure 7A**. The analysis of three groups of metabolites unraveled that the differential metabolites of HAD and VFD were mainly alcohols. At the same time, SD and HAD, SD and VFD were mainly esters and terpenes. Volcano plots demonstrated the relative content differences of metabolites in two groups of samples as well as their statistical significance. For each comparison group, the differential metabolites are shown in **Figure 7B–D**, respectively. Each point in the volcano plot represents a metabolite, where green points represent down-regulated differential metabolites, red points represent up-regulated differential metabolites, and gray represents the metabolites that showed no significant difference in the expression. The volcanic map illustrated that the differences were significant, and the screened differential metabolites were reliable.

**Figure 8:**
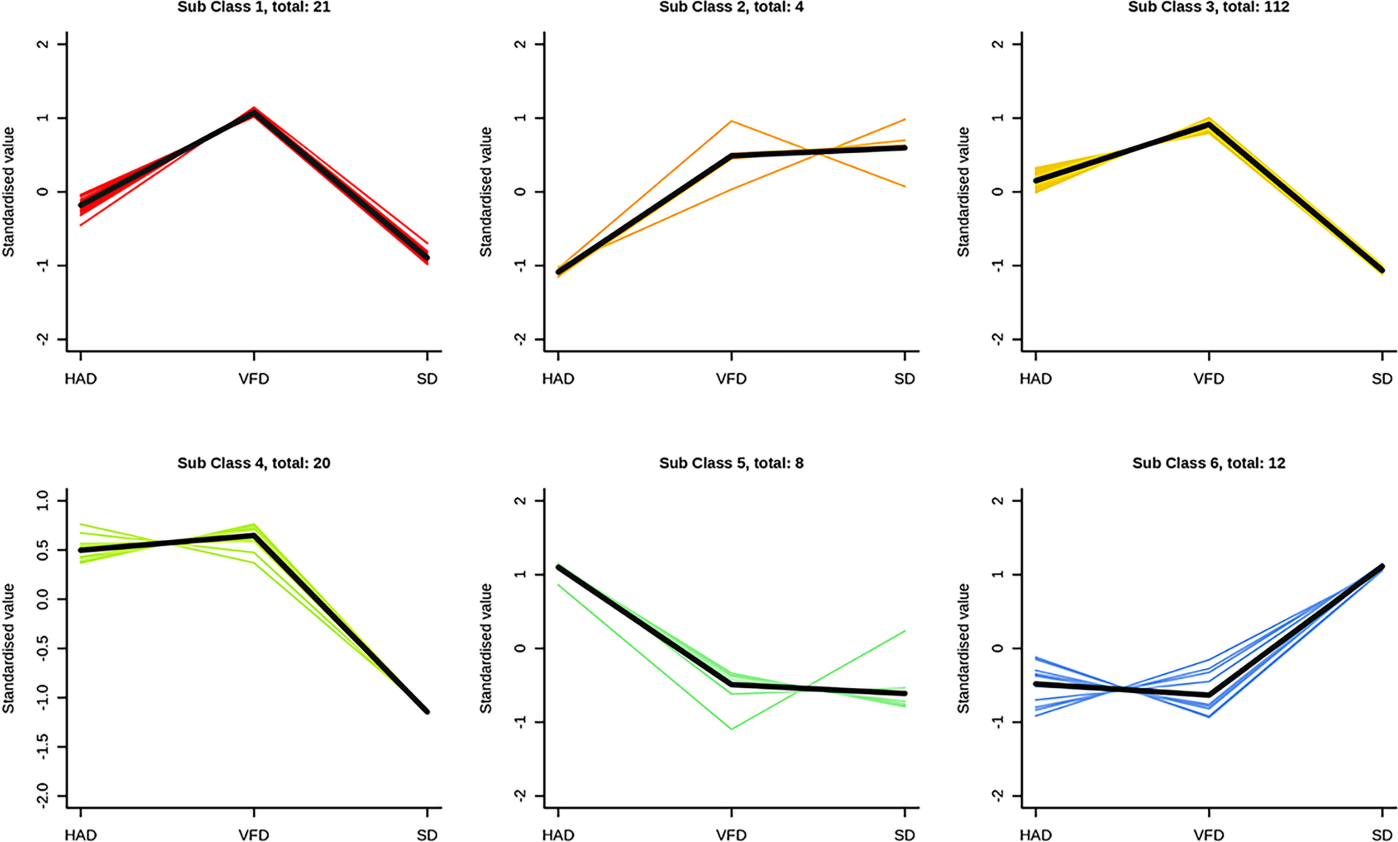
K-Means results of differential metabolites of CRCP with different drying methods. The abscess axis represents the sample name, the ordinate represents the relative content of standardized metabolites. The subclasses represent the number of metabolite classes with a similar trend. The total: *represents the number of metabolites in the class.

To investigate the trends of metabolites among different groups, the relative content of all differential metabolites in the comparison groups was normalized by Z-score, and the K-Means cluster analysis was performed as **Figure 8**. The differential metabolites were divided into six class using K-means clustering analysis. The relative content metabolite of the 5^th^ class was the highest in the SD sample, while the relative metabolite contents of the other five classes were the highest in the VFD sample. These results showed that HS-SPME-GC-MS is suitable for distinguishing and comparing CRCP samples derived using different drying methods. The analysis indicated that the relative content of VOCs in VFD samples was the highest, and the up-regulated differential metabolites were maximum in VFD sample. Overall, these results suggested that vacuum-freeze drying methods can effectively retain the content and type of VOCs in CRCP.

## 4 Discussion

Due to its distinctive aroma and flavor, CRCP has been utilized in traditional medicine and as a highly beneficial nutritional food source for centuries (Zhou et al., 2021). In China and around the world, CRCP is widely used in TCM, snacks, functional foods, tea, traditional spices, and condiments (Xiao et al., 2022). The uniqueness and irreplaceability of CRCP primarily depend on its variety, environment, climate, and production technology. A preliminary market survey showed that natural sun-drying is China′s primary drying method for CRCP production. Nevertheless, sun-dried CRCP faces the risk of being prone to moth-eaten and mildewing. Therefore, it is of utmost importance to identify a more efficient drying method for CRCP production to promote the development of the CRCP industry.

In this study, we used E-nose, GC-IMS, and HS-SPME-GC-MS approaches to compare VOCs in CRCP samples generated with different drying methods. About 101 VOCs were detected by GC-IMS in the CRCP samples with three drying methods, while 431 VOCs were detected by HS-SPME-GS-IMS analysis. The main reason for this difference could be that SPME has a strong trapping capacity for high-boiling compounds. Therefore, HS-SPME-GC-MS can detect more high-boiling compounds, and the content of high-boiling compounds is higher (Pu et al., 2019; Pu et al., 2020). In our study, the significant VOCs present in CRCP were terpenes and esters, consistent with the previous report (Duan et al., 2016). A comparison of the VOCs identified using GC-IMS in CRCP samples revealed that the SD contained the maximum types and highest contents of VOCs. However, a comparison by HS-SPME-GC-IMS showed that VFD exhibited the maximum VOCs, and almost all the differential metabolites were up-regulated compared to the other groups. The results of GC-IMS showed that sun-drying is the best drying method, consistent with the drying method used in the traditional production process of CRCP. However, the results of GC-IMS had some limitations, such as the types of compounds detected being incomplete. In the case of “methyl *n*-methylanthranilate”, a unique compound present in CRCP, as shown in the previous study, was undetected. In addition, 19 of the 101 VOCs detected could not be identified qualitatively. Next, 431 VOCs were detected by HS-SPME-GC-MS in CRCP samples with three drying methods, more than four times the number of GC-IMS. The detected VOCs included their unique volatile compounds as well as several terpenes. The HS-SPME-GC-IMS results indicated that vacuum-freeze drying is the best method for CRCP production.

With the increasing demand for high-quality foods and medicines, it is imperative to identify new herbs with better organic qualities such as texture and color without losing their nutritional or pharmacological properties. The vacuum-freeze drying method can preserve the product′s color, flavor, taste, shape, and nutritional components to the greatest possible extent (Xu et al., 2021). In addition, this process preserves the natural shape of the material without hardening its surface, and prolongs the storage period (Feng et al., 2020). Currently, vacuum-freeze drying has been widely used for various foods (Lu et al., 2022) and herbs (Yu et al., 2022). Currently, there is an excellent demand for CRCP in the market; however, the traditional sun-drying method requires a large area, and a long time, and it is highly susceptible to weather conditions. Therefore, to improve the production efficiency of CRCP, combined with the results of this study and the application of vacuum-freeze drying in the plant drying process, vacuum-freeze drying can be used as the drying method for large-scale drying of CRCP in the future. Vacuum-freeze drying retains more VOCs and maintains better shape and color of the dried CRCP. Moreover, it can avoid the deterioration of CRCP such as moth-eaten and moldy during storage and assure the healthy quality of CRCP. This study would guide the drying and processing of CRCP and promote the development of the CRCP industry.

## 5 Conclusion

In the current study, we have compared the VOCs of CRCP samples with different drying methods using E-nose, GC-IMS, and HS-SPME-GC-MS and found that significant differences in the types and contents of VOCs in different CRCP samples. The types and contents of VOCs in CRCP prepared by vacuum-freeze drying were better than those prepared using the other two drying methods. Our study provides new ideas and directions for the drying and processing technology of CRCP production, which might help to shorten the drying time and improve the yield and quality of CRCP. In order to further evaluate the effects of different drying methods on the quality of CRCP, the flavonoids in CRCP with different drying methods can be compared in the future, which will promote the development of CRCP industry based on more comprehensive results.

## Data availability statement

The original contributions presented in the study are included in the article/**Supplementary Material**. Further inquiries can be directed to the corresponding authors.

## Author contributions

MW, FW and LC designed and coordinated the study. MW, XL, HD and HC collected the samples and performed all of the experiments. YL identified the samples and provided academic support. MW, XL, FW and LC analyzed the data. MW, FW and LC wrote the manuscript with input from all authors. All authors contributed to the article and approved the submitted version.

## Supplementary material

Supplementary Figure 1 Working principle diagram of the E-nose.

Supplementary Figure 2 Working principle diagram of GC-IMS.

Supplementary Figure 3 TIC overlapping map of quality control sample (A) and TIC map of mixed sample (B)

Supplementary Figure 4 Chromatographic integration diagrams of the top ten VOCs with high content in CRCP

Supplementary Table 1 Volatile flavor compounds in CRCP with different drying methods by HS-SPME-GC-MS.

Supplementary Table 2 Differential metabolites in each comparison group.
