# Supplementary figures and images for: Comparison of the volatile organic compounds in Citrus reticulata ‘Chachi’ peel with different drying methods using E-nose, GC-IMS and HS-SPME-GC-MS
Source: Front Plant Sci. 2023 May 17;14:1169321. doi: 10.3389/fpls.2023.1169321 (PMC10231685; doi:10.3389/fpls.2023.1169321)

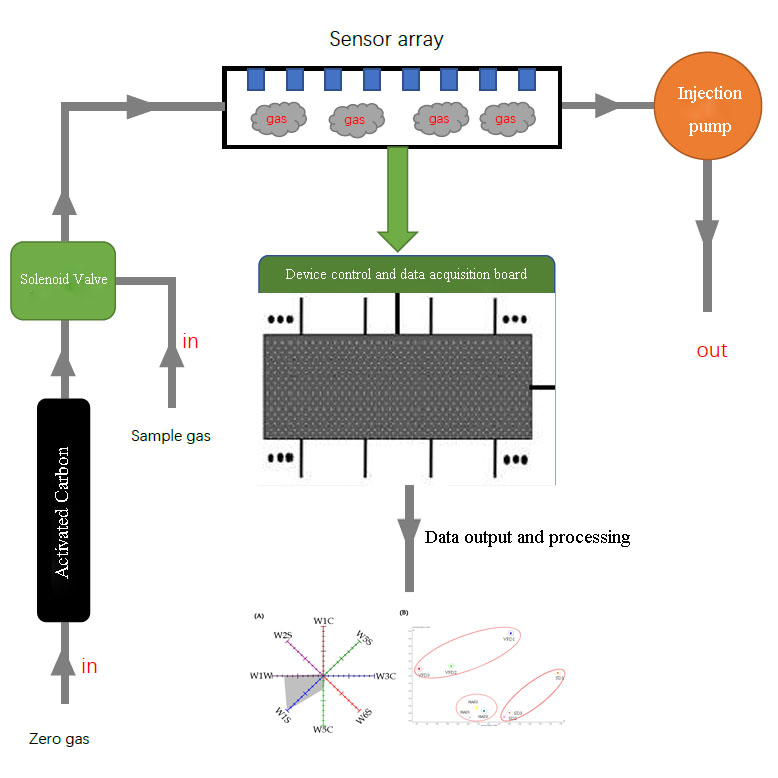

Supplement: Supplementary Figure 1 — Working principle diagram of the E-nose. [file Image_1.jpeg]

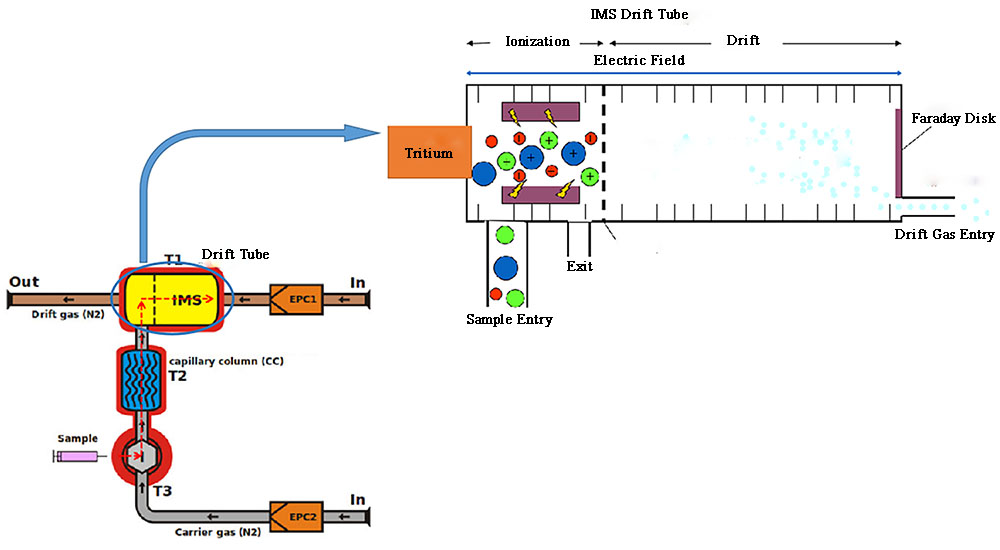

Supplement: Supplementary Figure 2 — Working principle diagram of GC-IMS. [file Image_2.jpeg]

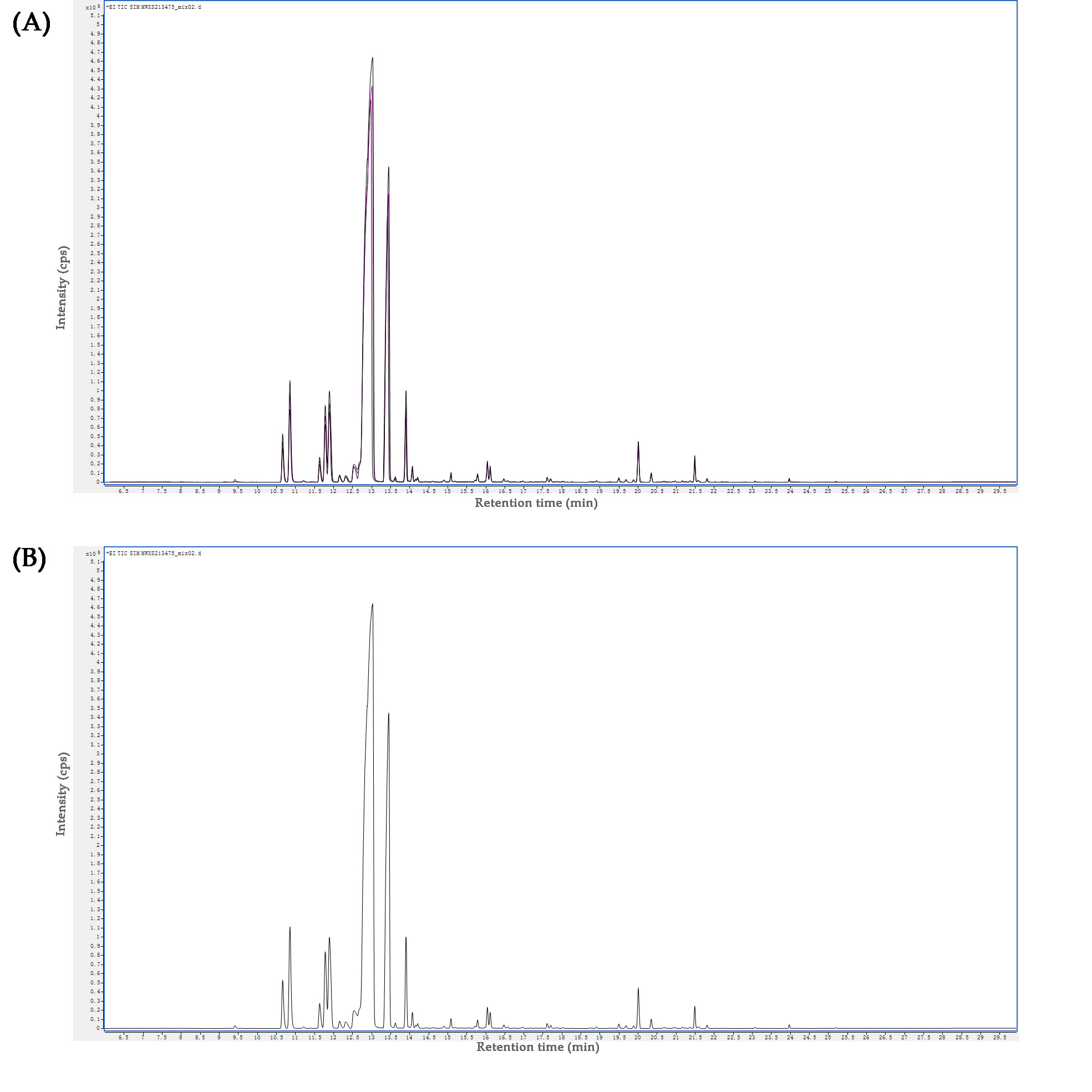

Supplement: Supplementary Figure 3 — TIC overlapping map of quality control sample (A) and TIC map of mixed sample (B) [file Image_3.jpeg]
