# Supplementary material for: Comparison of the volatile organic compounds in Citrus reticulata ‘Chachi’ peel with different drying methods using E-nose, GC-IMS and HS-SPME-GC-MS
Source: Front Plant Sci. 2023 May 17;14:1169321. doi: 10.3389/fpls.2023.1169321 (PMC10231685; doi:10.3389/fpls.2023.1169321)
Supplement: Supplementary Figure 4 — Chromatographic integration diagrams of the top ten VOCs with high content in CRCP [file DataSheet_1.docx]

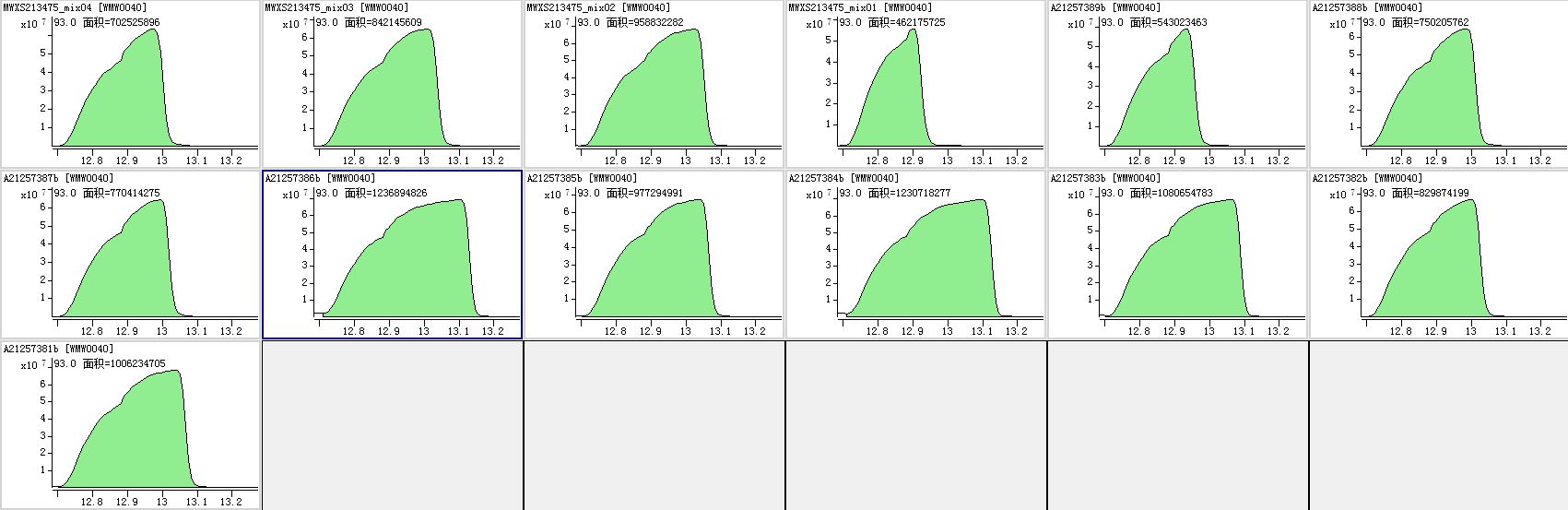


trans-.beta.-Ocimene


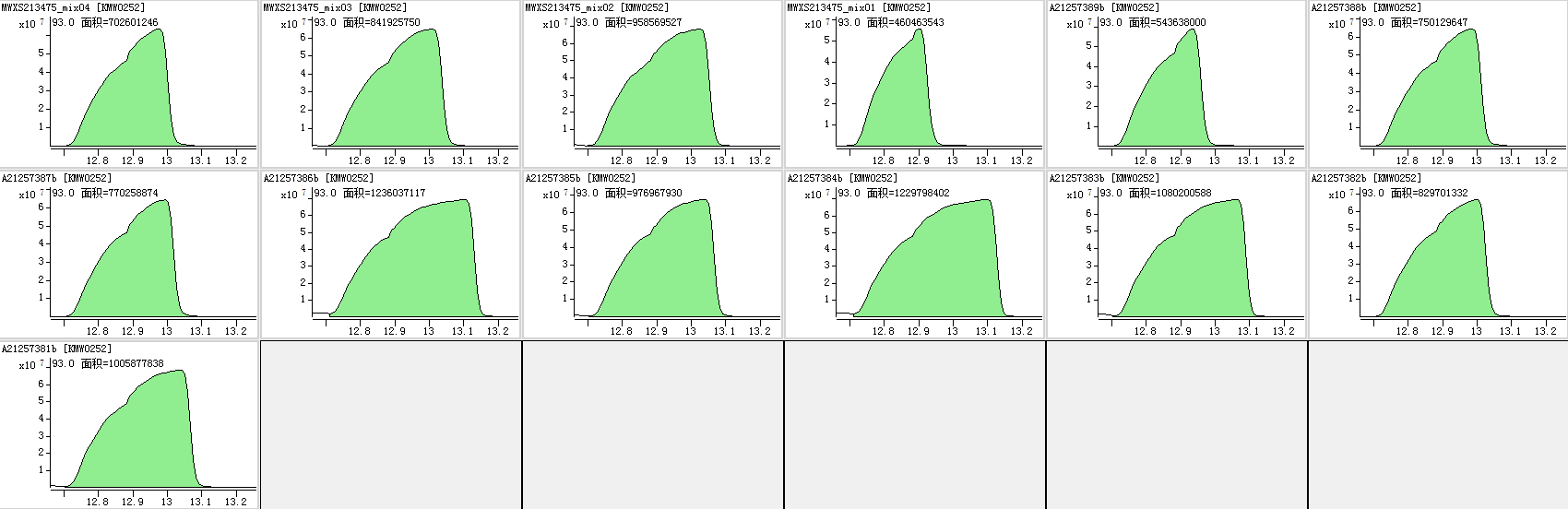


Alpha-Ocimene


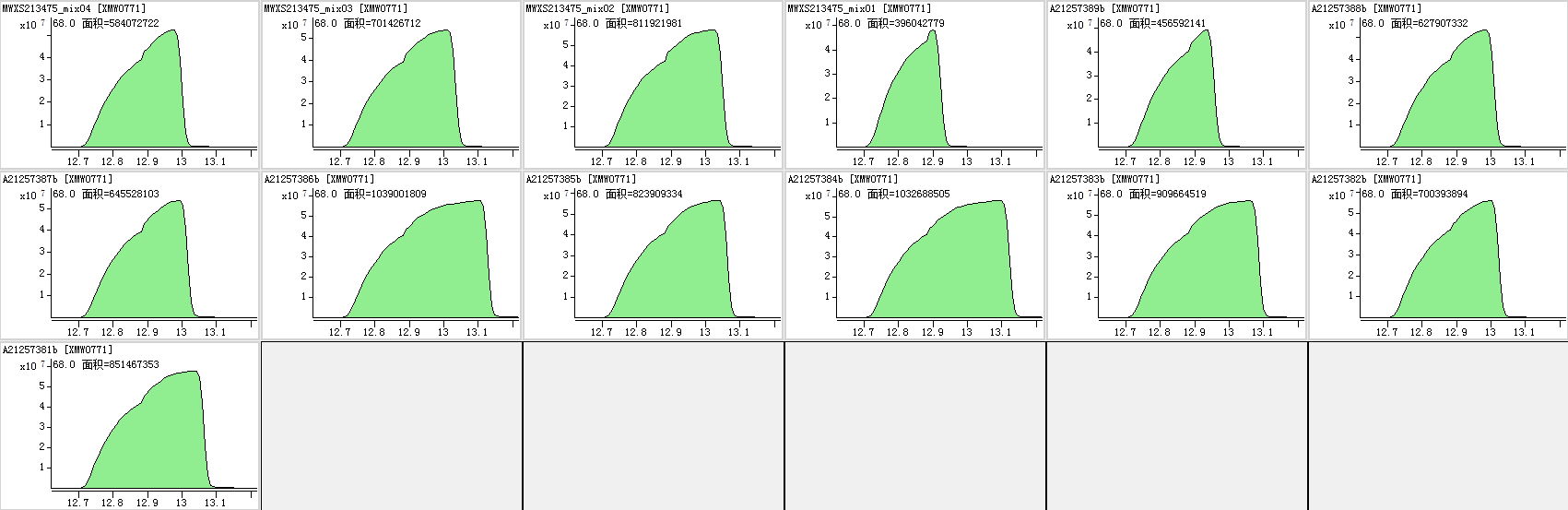


1,5-Cyclooctadiene, 3,4-dimethyl-


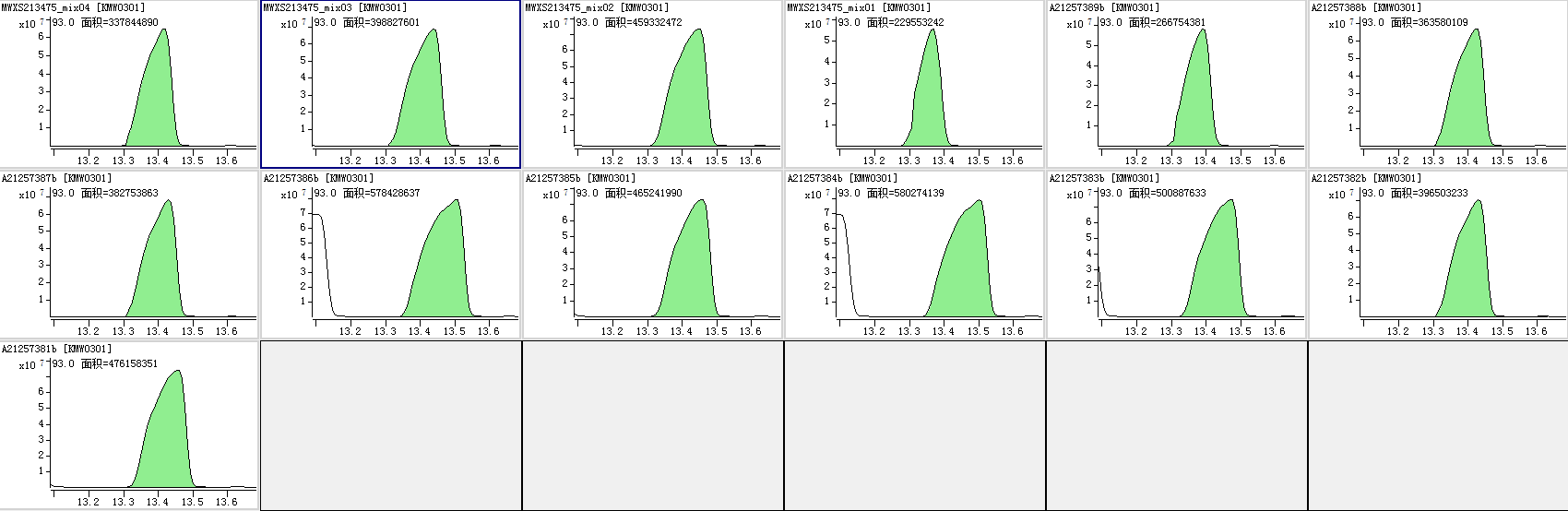


(E)-sabinene hydrate


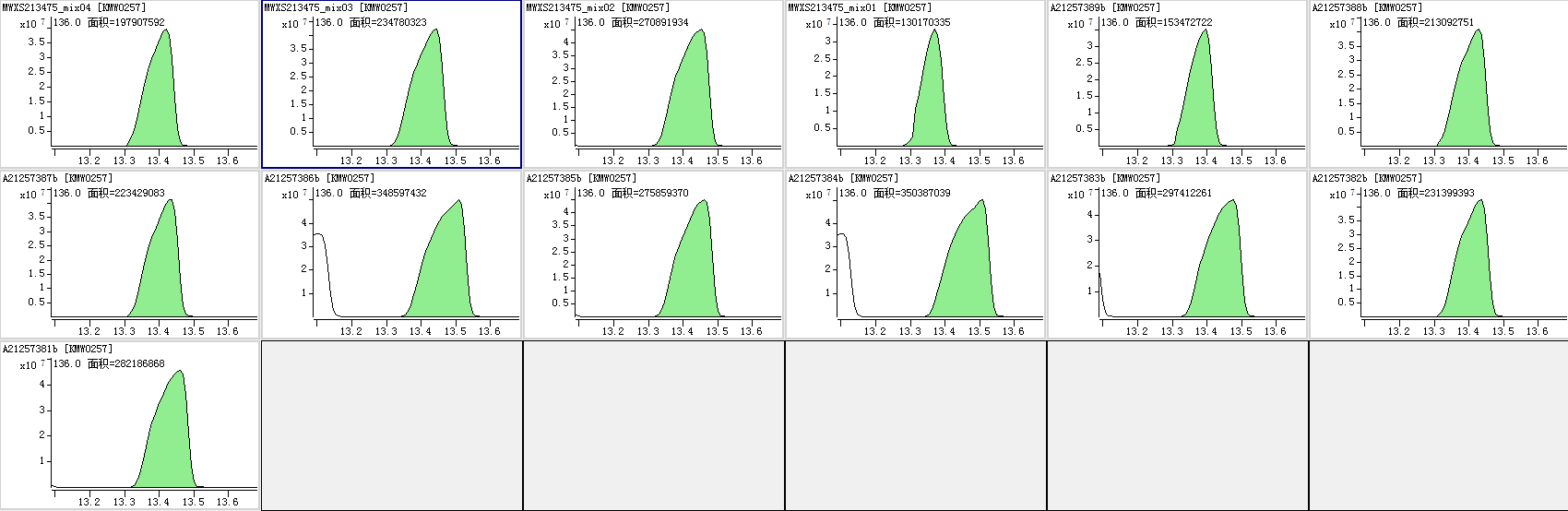


(Z)-sabinene hydrate


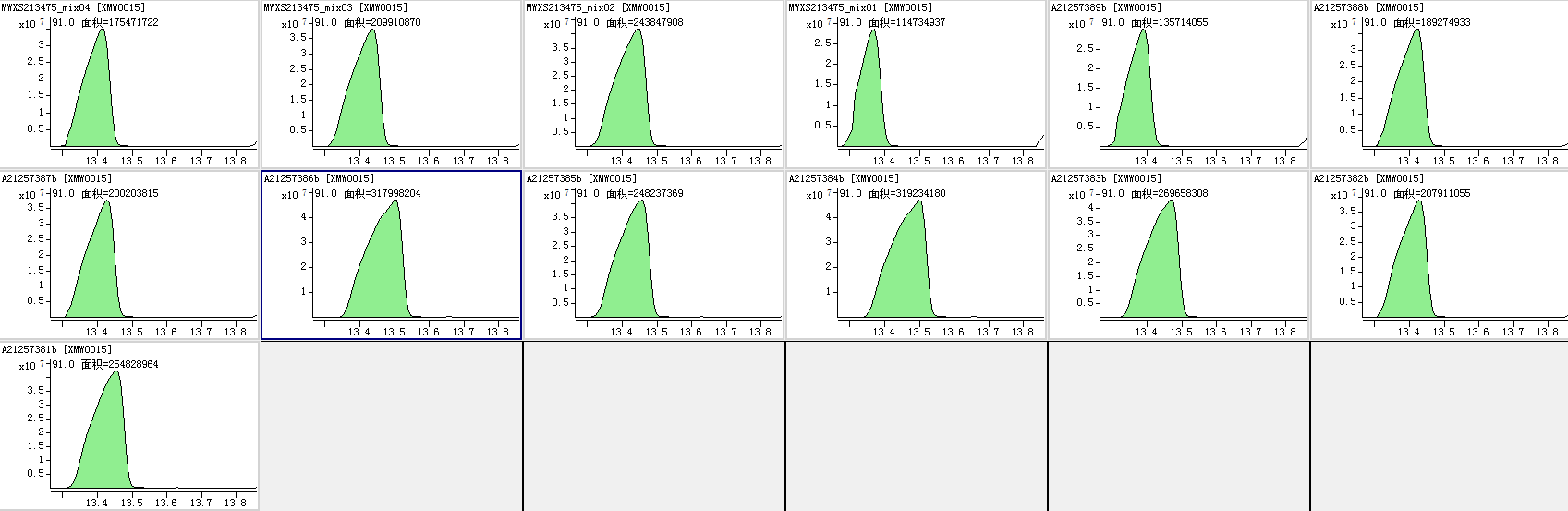


BenzAldehyde, 4-methyl-


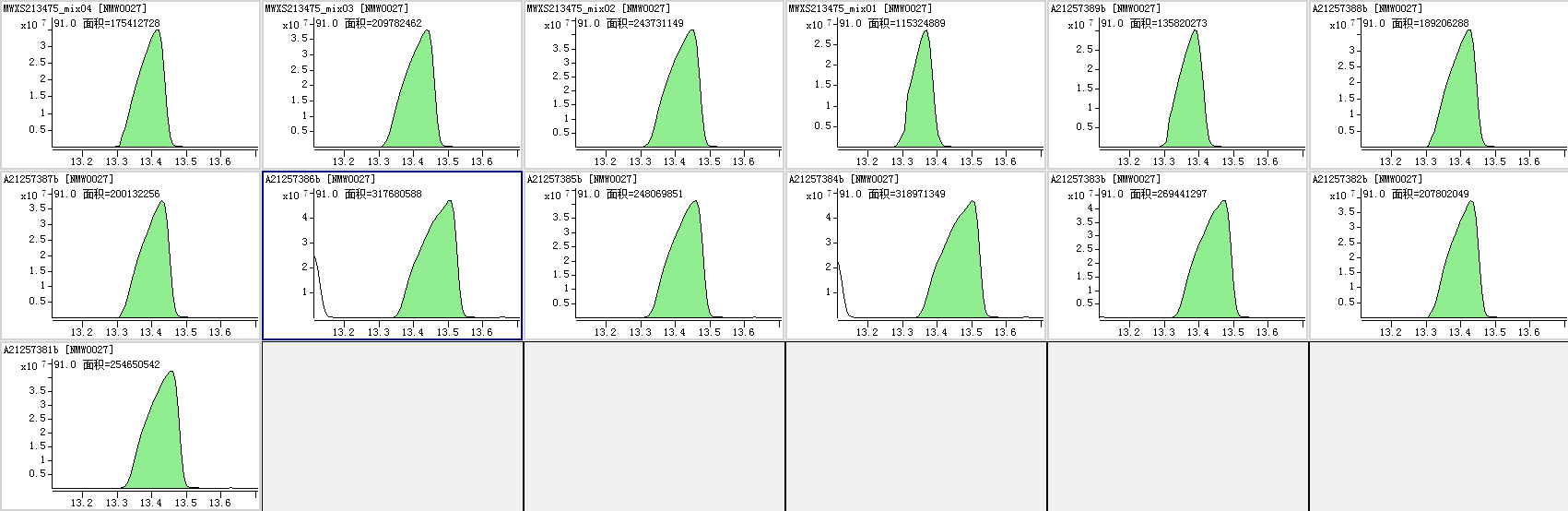


BenzAldehyde, 3-methyl-


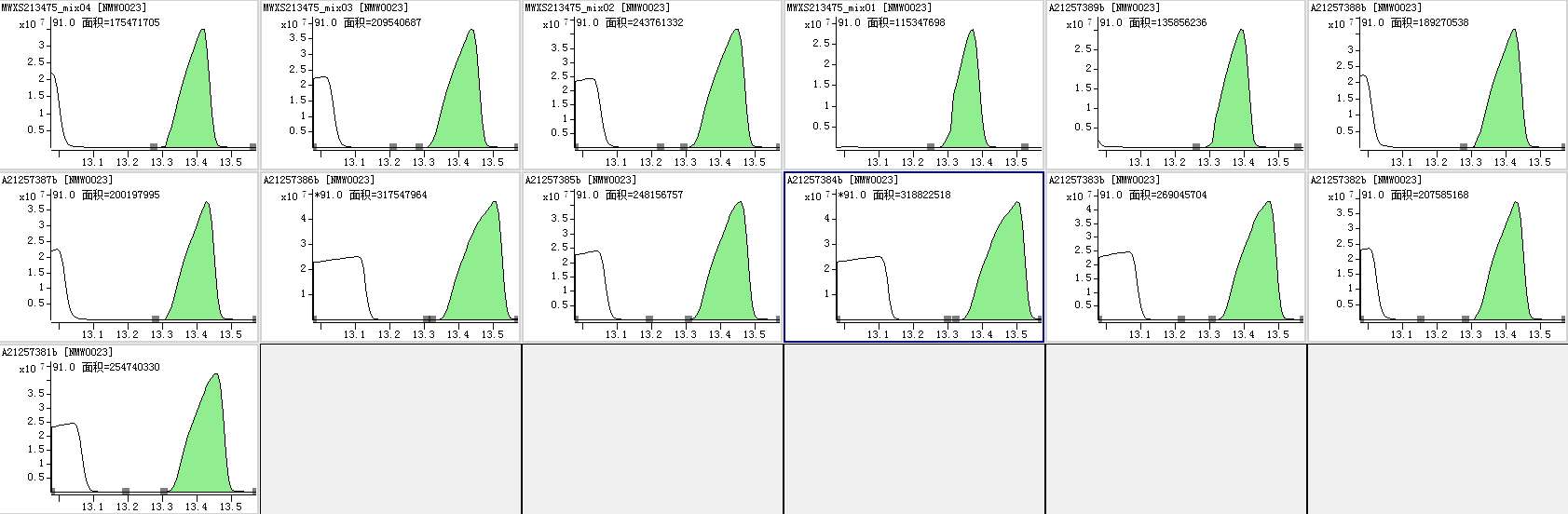


BenzAldehyde, 2-methyl-


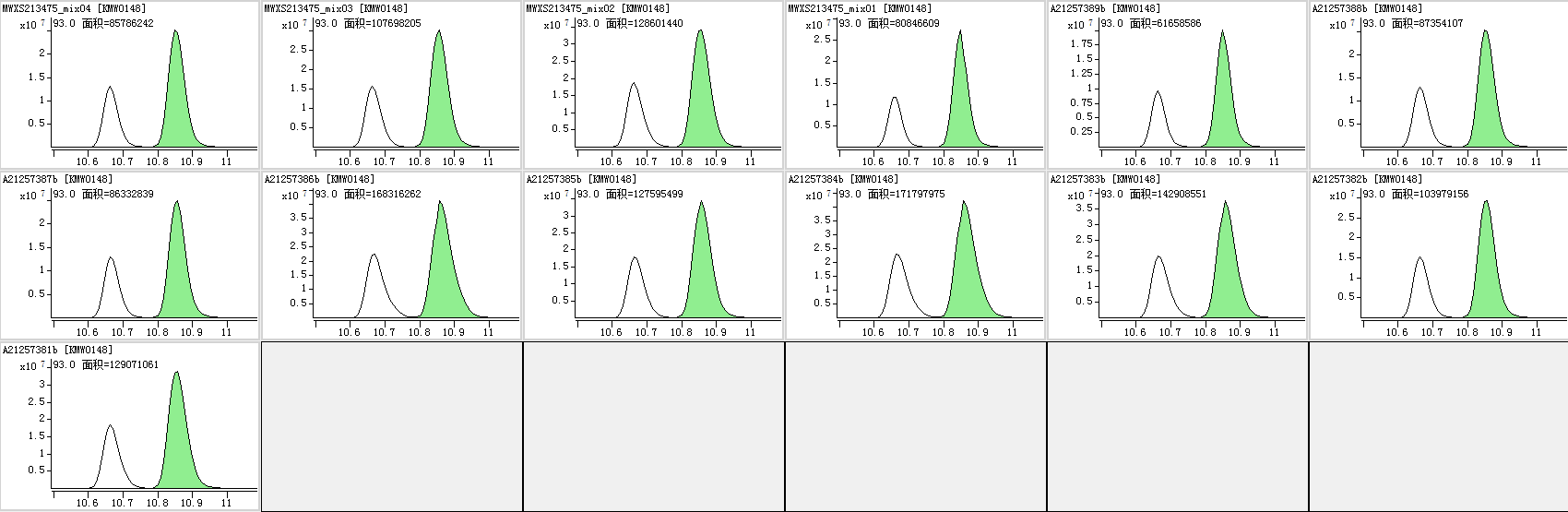


.alpha.-Pinene


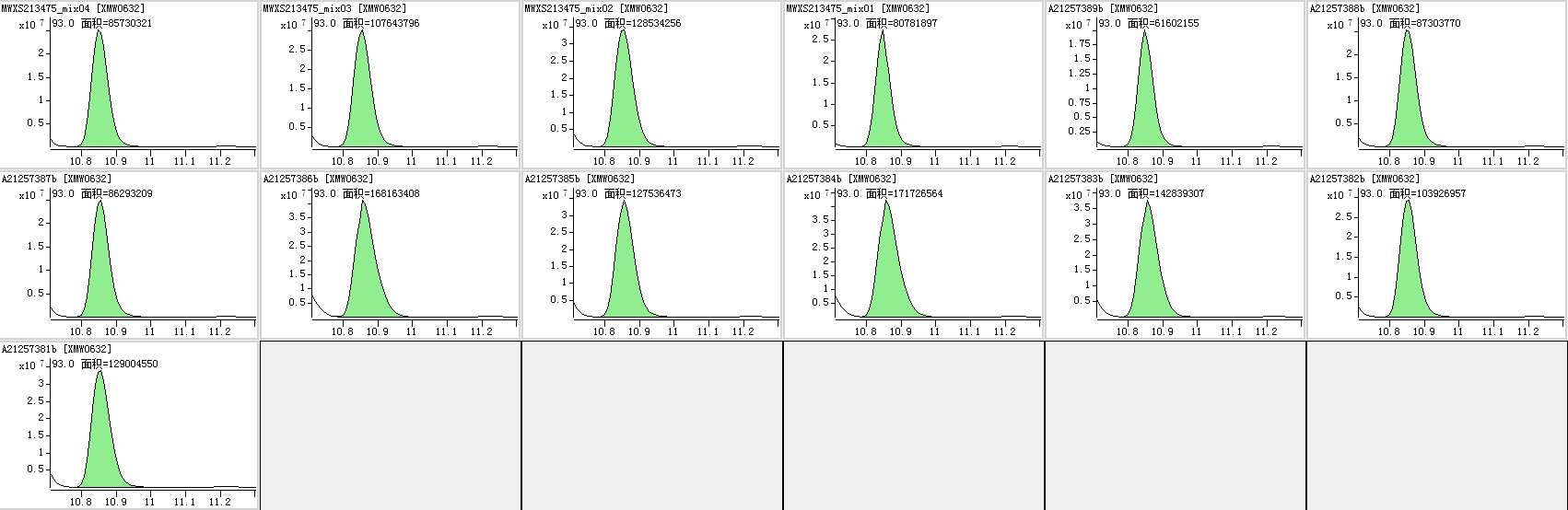


Bicyclo[3.1.1]hept-2-ene, 3,6,6-trimethyl-
